# Extracapsular nodal extension and tumor deposits in head and neck squamous cell carcinoma

**DOI:** 10.1002/cnr2.1897

**Published:** 2023-09-12

**Authors:** Leyre González‐Vallejo, Javier Blanco‐Sainzdelamaza, Arrate Querejeta‐Ayerra, Carlos Chiesa‐Estomba

**Affiliations:** ^1^ Radiotherapy Department Donostia University Hospital San Sebastian Spain; ^2^ Medical University of Bialystok Bialystok Poland; ^3^ Department of Otorhinolaryngology and Head and Neck Surgery Donostia University Hospital San Sebastian Spain

**Keywords:** extranodal extension, head and neck squamous cell carcinoma, pN3b classification, prognosis, tumor deposits

## Abstract

**Background:**

Tumor deposits (TDs) are an infrequently mentioned feature of head and neck squamous cell carcinoma (HNSCC) that are currently grouped under extranodal extension (ENE) in the AJCC 8th edition of HNSCC TNM staging. The prognostic implication of TDs in comparison to ENE remains uncertain.

**Methods:**

This observational, retrospective, non‐randomized study evaluated patients with HNSCC who underwent initial surgical resection, with neck dissection and adjuvant radiotherapy ± chemotherapy. Clinical variables were considered, and statistical analyses were conducted to compare time progression and overall survival (OS) in patients with TDs against those with ENE.

**Results:**

Of the 71 patients included in the study, 50 were diagnosed with ENE (pN2a‐ENE in 38 patients and pN3b‐ENE in 12), while 21 had TDs ± ENE. The median time to progression was significantly different based on the presence of ENE or TDs (*p* = .002) and pN2a‐ENE/pN3b‐ENE or TDs (*p* = .007). The three‐year OS was 55.7% for the entire group, 60.4% in ENE and 38.4% in TDs (*p* = .021). The OS difference between the pN2a‐ENE, pN3b‐ENE, and the TDs group was also significant (*p* = .05). The hazard ratio between ENE and TDs was Exp (B) 4.341 (*p* = .044).

**Conclusions:**

TDs in HNSCC are associated with a lower OS than ENE, despite intensified adjuvant therapy. Our results confirm a better prognosis for pN2a‐ENE vs. pN3b‐ENE, and pN3b‐ENE vs. TDs. TDs may serve as an indicator of poor prognosis and require separate TNM classification in HNSCC staging. Larger studies are needed to evaluate TDs impact on treatment strategies and outcomes.

## 1 INTRODUCTION

Cervical lymph node (LN) metastasis is an adverse prognostic factor in patients with head and neck squamous cell carcinoma (HNSCC). Furthermore, extranodal extension (ENE) is linked to a higher recurrence rate and distant metastasis (DM), and as a consequence, it has a negative prognostic value regarding the 5‐year overall survival (OS) rate.^1^, ^2^, ^3^, ^4^, ^5^

The eighth edition of the American Joint Committee on Cancer (AJCC) Cancer Staging Manual and the UICC TNM Classification of Malignant Tumors made substantial changes to the classifications of different head and neck (H&N) tumors.^6^, ^7^ ENE is now considered for both clinical N (cN) and postoperative N (pN) classifications, except for p16‐positive oropharynx, nasopharynx, and thyroid cancers. In addition, ENE is categorized as pN2a or pN3b, depending on the number and size of LNs. While these inclusions in TNM staging have reinforced the differentiation among the stages, there is likely room for further classification of different risk groups.

Tumor deposits (TDs) are defined as cancerous nodules composed of aggregates of tumor cells unrelated to locoregional LNs. Although TDs are included in the extracapsular group in the latest AJCC TNM staging, their significance as a prognostic indicator has been widely acknowledged in the literature.^8^, ^9^, ^10^, ^11^, ^12^, ^13^, ^14^, ^15^, ^16^ It is noteworthy that TDs are classified as a separate entity in some TNM staging tumors; for example, their presence in colorectal cancer is classified as N1c.^17^

The aim of this study is to detect the presence of TDs in HNSCC samples and analyze their association with other clinical variables, OS, and disease progression, compared to cases where ENE is present.

## 2 MATERIALS AND METHODS

### 2.1 Patients

We conducted an observational, retrospective, non‐randomized study after obtaining approval from the ethics committee of Donostia University Hospital (AVV‐EDC‐2017‐01) on patients with HNSCC staged according to the 8th Edition criteria of the AJCC. We selected patients aged 18 years or older who underwent initial surgery with neck dissection (ND) followed by adjuvant radiotherapy (RT) ± chemotherapy (CTX) between 2012 and 2018, while excluding those with previous ND, RT, or chemoradiation (CTX‐RT). Moreover, patients with synchronous malignancy, nasopharyngeal cancer, less than 3 years of follow‐up, HNSCC of unknown primary and non‐squamous cell histologies were also excluded. Adjuvant treatment was indicated according to the National Comprehensive Cancer Network (NCCN) guidelines.^18^ The postoperative histopathological findings were evaluated by pathologists following the institutional protocol. ENE and TDs were considered independent entities. Patients with ENE ± TDs were classified as having TDs since TDs were considered as a higher potential prognostic impact. We examined age (≤60 years/>60 years), gender, location (lips or oral cavity/salivary glands/hypopharynx/oropharynx/larynx), T category (T1/T2/T3/T4), N category (N ≤ 3 cm + ENE/N > 3 cm + ENE/TD), systemic treatment, type of progression (locoregional/distant), time to progression (in months), and OS at 3 years. OS was defined as the period between the date of surgery and the date of death or last follow‐up, measured in months.

We used IBM SPSS v26 software for statistical analysis. The Kolmogorov–Smirnov and Shapiro–Wilk tests were performed to evaluate the absence of normal data distribution, and nonparametric tests were used. The Mann–Whitney U test and Kruskal‐Wallis 1‐way ANOVA tests examined the differences in medians among groups for two samples and more than two samples, respectively. In order to investigate the relationship between variables in the various groups, we employed a Multinomial logistic regression test and calculated the Likelihood Ratio (LRT). Prior to this analysis, to ensure the validity of our results we assessed multicollinearity for each predictor variable by calculating variance inflation factor (VIF) values using a linear logistic regression (independent variables if VIF = 1; variables moderately correlated if VIF 1 < VIF <5; multicollinearity among the predictors if VIF ≥5 to 10).

Survival was calculated by the Kaplan–Meier method, and the log‐rank test was employed to compare survival curves. A univariate Cox regression analysis using the enter method was conducted, evaluating the correlation between OS and clinical factors, including gender, age, tumor location, T category, systemic treatment, local‐regional progression and the presence of ENE or TDs. A multivariate Cox regression analysis using the enter method was performed to simultaneously assess the effect of the risk factors on OS and to estimate the corresponding hazard ratios (HRs). Additionally, since including patients with both ENE and TD simultaneously could be considered a confounding factor, we performed a sensitivity analysis by excluding patients who had both ENE and TDs. After removing these patients, we repeated all the statistical tests to ensure the robustness of our results. In all cases, a statistically significant test result was considered as *p* < .05.

## 3 RESULTS

### 3.1 Patients' characteristics

This study analyzed the medical records of 71 patients who met the inclusion criteria and received treatment at the Department of Radiotherapy of Donostia University Hospital. Of the patients examined, 36 (50.7%) underwent bilateral dissection, while 35 (49.3%) underwent unilateral dissection. A majority of the patients were men (80.3%) and the population above the age of 60 was 59.2%. The most common primary site was found to be the oral cavity (35.2%), followed by the larynx (25.4%). Nearly half of the patients had T4 tumors, while approximately 40% had T1‐T2 tumors. Among the 71 patients, 21 (29.6%) were found to have TDs (6 TDs + ENE and 15 TDs without ENE), whereas 50 patients had ENE, comprising of 38 pN2a‐ENE (53.5%) and 12 pN3b‐ENE (16.9%). VIF values indicated that collinearity was not a significant concern (Table 1).

**TABLE 1:** Patient and tumor characteristics (*n* = 71).

	*N* (%)	pN2a‐ENE, *N* (%)	pN3b‐ENE, *N* (%)	TD, *N* (%)	VIF	LRT	Significance
Gender					1.101	0.462	0.192^a^
Male	57 (80.3)	28 (49.1)	11 (19.3)	18 (31.6)			
Female	14 (19.7)	10 (71.4)	1 (7.1)	3 (21.4)			
Age					1.135	0.134	0.303^a^
≤60	29 (40.8)	17 (58.6)	6 (20.7)	6 (20.7)			
>60	42 (59.2)	21 (50.0)	6 (14.3)	15 (35.7)			
Location					1.093	0.252	0.279^b^
Lips/oral cavity	25 (35.2)	15 (60.0)	2 (8.0)	8 (32.0)			
Salivary glands	4 (5.6)	3 (75.0)	0	1 (25.0)			
Hypopharynx	10 (14.1)	3 (30.0)	2 (20.0)	5 (50.0)			
Oropharynx	14 (19.7)	9 (64.3)	4 (28.6)	1 (7.1)			
Larynx	18 (25.4)	8 (44.4)	4 (22.2)	6 (33.3)			
T					1.073	0.918	0.730^b^
T1	12 (16.9)	5 (41.7)	3 (25.0)	4 (33.3)			
T2	17 (23.9)	10 (58.8)	4 (23.5)	3 (17.6)			
T3	8 (11.3)	5 (62.5)	1 (12.5)	2 (25.0)			
T4	34 (47.9)	18 (52.9)	4 (11.8)	12 (35.3)			
Systemic treatment					1.207	0.744	0.285^a^
Yes	58 (81.7)	29 (50.0)	11 (19.0)	18 (31.0)			
CBDCA	2 (2.8)	1 (50.0)	0	1 (50.0)			
CDDP	41 (57.7)	20 (48.8)	9 (22.0)	12 (29.3)			
Cetuximab	15 (21.1)	8 (53.3)	2 (13.3)	5 (33.3)			
No	13 (18.3)	9 (69.2)	1 (7.7)	3 (23.1)			
Spread					1.035	0.098	0.328^b^
No	36 (50.7)	20 (55.6)	6 (16.7)	10 (27.8)			
Local‐regional	11 (33.8)	7 (63.6)	3 (27.3)	1 (9.1)			
Distant	24 (15.5)	11 (45.8)	3 (12.5)	10 (41.7)			
Total		38 (53.5)	12 (16.9)	21 (29.6)			

Abbreviations: ENE, extranodal extension; LRT, likelihood ratio tests significance; TD, tumor deposit; VIF, variance inflation factor.

^a^ Mann–Whitney U (2 independent samples).

^b^ Kruskal‐Wallis 1‐way ANOVA (*k* independent samples).

Intensity‐modulated radiotherapy was administered at five fractions per week. The treatment began between 4 and 8 weeks after surgery in all cases. The median dose prescription was 64.5 Gy for high‐risk regions, administered in 30 fractions (radiation biologically effective dose: 78.37 Gy). Concomitant Cisplatin‐based chemotherapy (CTX) was given to 57.7% of patients, while 21.1% of patients received Cetuximab. Only two cases (2.8%) were treated with Carboplatin‐based CTX (CBDCA), and 18.3% of patients did not receive systemic treatment.

### 3.2 Outcomes

The overall progression rate was 49.3% (Table 2) with a median time to progression of 13 months. Based on the presence of ENE alone or TDs, the median time to progression was 14 months in the group with ENE and 9 months in the TDs group, with the difference being statistically significant (*p* = .002). Conversely, based on their *N* classification, the median time to progression was 13 months in the pN2a‐ENE group and 15 months in the pN3b‐ENE group (*p* = .007). Of the patients who progressed, 11 developed locoregional recurrences (31.4%), and DM was diagnosed in 24 (68.6%) without any significant differences in the progression time. Most patients who progressed were male (82.9%), 60% were older than 60 years, and 40% had oral cavity tumors. Regarding the T category, 51.4% of patients who progressed in the disease had T4 tumors, while 22.9% of the cases were T1 tumors with a longer median time to progression in T1‐T2 than in the advanced groups (*p* = .047). Most of the patients who progressed received systemic therapy (82.9%). From the cases that progressed, 61.11% (11/18) of patients in the pN2a‐ENE subgroup and 50% (3/6) of patients in the pN3b group exhibited DM. Remarkably, in the TDs group, 90.91% (10/11) of patients who experienced progression developed DM (Table 1).

**TABLE 2:** Characteristics and time to the progression.

	*N* (%)	Median time to progression (months)	95% CI	Sig.
Progression				
No	36 (50.7)			
Yes	35 (49.3)	13	9.557–16.443	
Gender				0.082
Male	29 (82.9)	13	9.101–16.899	
Female	6 (17.1)	12	0.000–24.002	
Age				0.431
≤60 years	14 (40.0)	14	11.555–16.445	
>60 years	21 (60.0)	10	7.778–12.222	
Location				0.466
Lips/oral cavity	14 (40.0)	12	8.333–15.667	
Salivary glands	3 (8.6)	13	6.599–19.401	
Hypopharynx	6 (17.1)	10	6.399–13.601	
Oropharynx	5 (14.3)	19	10.412–27.588	
Larynx	7 (20.0)	8	2.868–13.132	
T				0.047
T1	8 (22.9)	14	2.913–25.087	
T2	5 (14.3)	18	13.706–22.294	
T3	4 (11.4)	9	0.180–17.820	
T4	18 (51.4)	9	6.228–11.772	
Systemic treatment				0.697
Yes	29 (82.9)	12	8.044–15.956	
No	6 (17.1)	13	8.999–17.001	
Spread				0.530
Local‐regional	11 (31.4)	12	8.159–15.841	
Distant	24 (68.6)	14	9.145–18.855	
ENE/TD				0.002
ENE	24 (68.6)	14	11 608‐16 392	
TD	11 (31.4)	9	6.572–11.428	
pN2a‐ENE/pN3b‐ENE/TD				0.007
pN2a‐ENE	18 (51.4)	13	8.842–17.158	
pN3b‐ENE	6 (17.1)	15	12.600–17.400	
TD	11 (31.4)	9	6.572–11.428	

Abbreviations: CI, confidence interval; ENE, extranodal extension; Sig, log rank (Mantel‐Cox) significance; TD, tumor deposit.

**FIGURE 1:**
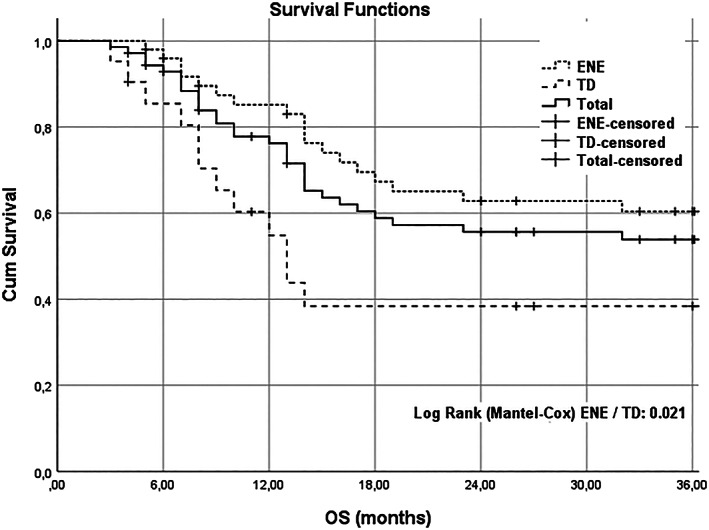
Kaplan–Meier plots. Overall Survival and differences between extranodal extension (ENE) and tumor deposits (TDs) groups.

**FIGURE 2:**
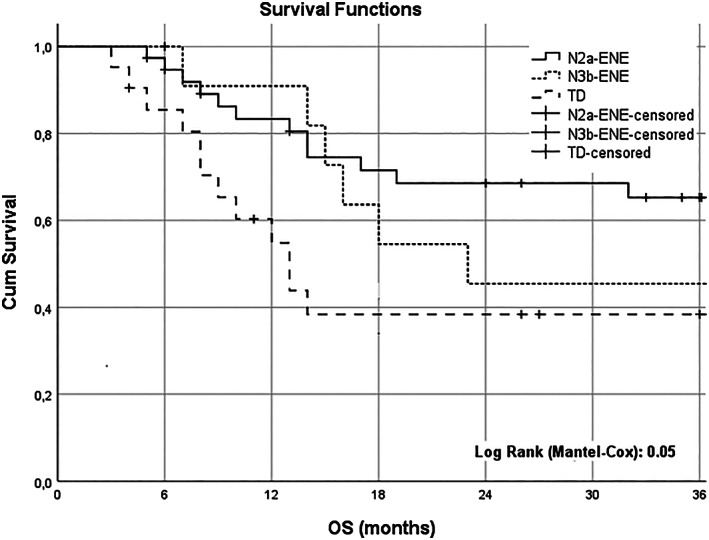
Kaplan–Meier plots. Overall survival (OS) for groups with extranodal extension (ENE) in pN2a, pN3b, and tumor deposits (TDs).

The OS rate at 3 years was 55.7% for the whole study group, 60.4% in ENE, and 38.4% in TDs (*p* = .021) (Figure 1). The presence of TDs was found to have a significant association with OS in the univariate analysis, with a HR of 0.436 (95% confidence interval [CI]: 0.209–0.909, *p* = .027) compared to those with ENE. However, no significant association was observed between OS and gender, age, location, T classification, systemic treatment, or spread. After adjusting for other risk factors, TDs remained a significant factor associated with OS in the multivariate analysis, with a HR of 4.341 (95% CI: 1.038–18.158, *p* = .044) compared to those with ENE. None of the other risk factors were found to be significantly associated with OS in the multivariate analysis (Table 3). The OS at 3 years was found to be 65.3% for the group with pN2a, 45.5% for the group with pN3b, and 38.4% for the group with TDs. (log rank Mantel‐Cox significance: 0.05) (Figure 2).

**TABLE 3:** Overall survival in ENE and TDs.

	OS			
	Univariate analyses		Multivariate analyses	
	HR (95% CI)	*p*	HR (95% CI)	*p*
Gender	0.781 (0.299–2.042)	.614	0.778 (0.169–3.586)	.747
Male				
Female				
Age	1.892 (0.884–4.051)	.100	0.908 (0.326–2.528)	.853
≤60				
>60				
Location	1.047 (0.800–1.371)	.736	1.107 (0.758–1.618)	.598
Lips/oral cavity				
Salivary glands				
Hypopharynx				
Oropharynx				
Larynx				
T	1.285 (0.931–1.775)	.128	1.202 (0.771–1.874)	.417
T1				
T2				
T3				
T4				
Systemic treatment	1.431 (0.614–3.337)	.406	1.481 (0.318–6.904)	.617
Yes				
CBDCA				
CDDP				
Cetuximab				
No				
Spread	0.822 (0.350–1.927)	.652	1.144 (0.424–3.085)	.791
Local‐regional				
Distant				
ENE/TD	0.436 (0.209–0.909)	.027	4.341 (1.038–18.158)	.044
ENE				
TD				

Abbreviations: CI, confidence interval; ENE, extranodal extension; HR, hazard ratio; OS, overall survival; *p*, significance; TD, tumor deposit.

In order to assess the impact of patients with both ENE and TDs on our findings, a sensitivity analysis was performed by excluding six patients from the study. After their exclusion, the univariate Cox regression analysis still yielded significant results for ENE/TDs (Exp (B) 0.439, 95% CI 0.196–0.982; *p* = .045). In the multivariate analysis, the presence of implants was found to have a non‐significant effect on OS (Exp (B) 0.263, 95% CI 0.059–1168; *p* = .079). Other variables, including age, sex, primary tumor number, T stage, systemic treatment, and LN ratio, did not demonstrate significant associations with OS in the multivariate analysis. According to the Kaplan–Meier estimator, the OS for the groups with ENE and TDs was significant (*p* = .037). While some of our results showed changes after excluding these cases, the overall conclusions of our study remained largely consistent, indicating that the presence of both ENE and TDs had minimal impact on our findings.

## 4 DISCUSSION

Extranodal extension in HNSCC has long been recognized as a poor prognostic factor since 1970.^19^ It is diagnosed based on criteria established by the College of American Pathologists, indicating tumor extension beyond the LN capsule, with or without a desmoplastic stromal reaction.^20^ The recent eighth edition of the AJCC staging manual introduced significant changes to the classification of H&N tumors, categorizing ENE as pN2a or pN3b based on the number and size of LNs in HPV‐negative HNSCC.^6^, ^7^, ^21^ Alongside ENE, the presence of TDs serves as another prognostic indicator in HNSCC.^8^ TDs are characterized as free soft tissue deposits that lack apparent continuity with the primary carcinoma cells and show no discernible organized lymphoid tissue at their periphery.^9^ The Lewis 4‐point grading system assigns grade 4 to ENE, aligning with the description of TDs.^22^ Nevertheless, further validation is required to determine if TDs represent a distinct entity from ENE or indicate LN replacement by the tumor, rendering the surrounding LN tissue unrecognizable.^12^

In our study, encompassing a series of HNSCC cases, approximately 50% of the cases were classified as pN2a, 16.9% as pN3b, and TDs were observed in 29.6% of cases (21 patients), slightly higher than reported in other studies. However, TDs are often compared with involved nodes, and their comparison with ENE is less frequent, as they are typically analyzed together.^11^, ^12^, ^13^

ENE has been associated with advanced T stages in HNSCC; however, our findings reveal that it can also occur in smaller tumors. We found ENE to be present in 16.9% of T1 tumors and 23.9% of T2 tumors, aligning with previous studies conducted by Ghadjar et al., who reported ENE in 7% of T1 stage tumors,^23^ and Mair et al., who found ENE in 13.4% of T1 and 16.8% of T2 oral cancer patients.^24^ Additionally, ENE can be observed in LNs of varying sizes. Although ENE is commonly observed in LNs larger than 3 cm, it may also be present in smaller nodes measuring less than 1 cm.^23^, ^25^ Macroscopically visible extracapsularity has been identified as a clinically relevant adverse prognostic factor.^22^, ^26^, ^27^, ^28^ However, we did not analyze the differences between macroscopic and microscopic ENE cases, due to the uncertainty surrounding the prognostic significance of microscopic ENE in comparison to macroscopic ENE.^2^, ^11^, ^29^, ^30^ Further investigation is necessary to determine if aggressive treatment is only required for patients with macroscopic ENE.

The overall incidence of TDs in HNSCC ranges between 7% and 24% and they are more prevalent in advanced stages.^9^, ^12^, ^14^ In our study, 35.3% of TD‐associated tumors were categorized as T4 stage. In contrast, Ozmen et al. reported a significantly higher proportion, with 57% of TD‐associated tumors falling into the T4 stage category.^14^ Moreover, our research findings revealed that TDs were observed in 33.3% of T1 stage tumors and 17.6% of T2 stage tumors, indicating that TDs can be present in early‐stage tumors, much like the occurrence of ENE. Similarly, Malik et al. reported a prevalence of 29% of TDs in pT1‐T2 cases within their series on STDs.^15^ In contrast, the Jose et al. series found lower rates, with 11% of TDs in T2 tumors and 5% in T1 tumors.^12^ These variations in TD prevalence across the studies highlight the need for further research to understand the underlying factors influencing TDs presence in different tumor stages.

Disease recurrence within the first 3 years is the primary cause of death in patients with HNSCC.^31^ The presence of TDs can significantly impact disease‐free survival and OS, increasing the risk of both locoregional and distant recurrences,^10^, ^13^, ^14^ as demonstrated in our present study. Although previous studies have generally shown locoregional recurrences to be more common than DM in TDs,^13^, ^14^, ^15^ our study revealed an opposite trend. Specifically, our findings revealed that among patients with TDs who experienced tumor progression, 90.91% exhibited DM. These results suggest that even though a treatment approach of surgery followed by adjuvant CTX‐RT may reduce locoregional failure in patients with TDs, it does not prevent distant failure. Furthermore, among our patients, the median time to progression for locoregional failure was 12 months, while for distant failure, it was 14 months, though this difference did not reach statistical significance. Notably, the median time to progression varied significantly between pN2a‐ENE, pN3b‐ENE, and TDs, ranging from 13 months in pN2a‐ENE to 9 months in TDs, with a statistically significant difference. These findings further support the AJCC pN classification of HNSCC, which distinguishes between pN2a‐ENE and pN3b‐ENE, and highlight the potential benefit of incorporating TDs in the staging system to enhance prognostic accuracy.

Recent studies have reported that ENE is linked to an increased risk of mortality, recurrence, and DM in patients with HPV‐positive oropharyngeal squamous cell carcinoma (OPSCC).^32^, ^33^ Our study observed a higher frequency of cases in locations typically not associated with HPV, such as the oral cavity and larynx, therefore our study did not analyze the presence of HPV. In addition, due to the limited sample study, further classifications or sub‐classifications related to HPV status would not have significantly contributed to our study's objectives. However, the lack of negative predictive value of ENE among patients with HPV‐positive OPSCC observed in different studies resulted in its absence from their current staging (AJCC 8th edition).^34^, ^35^

In the present research, the estimated HR between ENE and TDs in a Cox multivariate analysis, was significant (Exp (B) 4.341; *p* = .044). This finding suggests that TDs are associated with a lower OS than ENE, which is consistent with results from previous studies. Sarioglu et al. found that TDs were associated with a 3.2‐fold reduction in OS,^13^ while Yu et al. reported that TDs were an independent predictor of poor prognosis, associated with decreased OS, disease‐specific survival and recurrence‐free survival rates according to univariable and multivariable analyses.^16^ However, the role of TDs as an independent prognostic value has yet to be confirmed, which may influence current practice in HNSCC.

When assessing the effect of TDs on survival, several authors consider ENE as a confounding factor, as the two are often analyzed together.^9^, ^12^ This is because both ENE and TDs can be observed in up to 50% of cases.^12^ While the presence of both entities may have an additive effect on prognosis, other factors such as the size and number of TDs, the number and size of affected nodes, or the presence of non‐extracapsular LN involvement (*N*‐status) may also influence this prognosis.

In our series, TDs remained the variable closest to significance in the multivariate analysis, even after excluding six patients with both ENE and TDs. This observation suggests that patients with TDs may have a lower likelihood of survival compared to those with ENE, indicating that TDs may possess some prognostic value, although the statistical significance did not reach the conventional threshold of 0.05. Moreover, it is important to highlight that further reducing our sample size could increase the risk of false‐negative results, potentially underestimating the prognostic value of TDs. Therefore, despite the Cox multivariate analysis not showing statistical significance after excluding cases with simultaneous ENE and TDs, TDs should still be considered as a potential prognostic factor in future studies with a larger sample size. These findings align with the research conducted by Violaris et al., who similarly demonstrated that patients with TDs have a worse prognosis compared to those with ENE, a conclusion supported by several subsequent studies.^10^, ^11^, ^12^

ENE can be observed in a significant proportion of metastatic LNs that are smaller than 1 cm, indicating the aggressive nature of the primary tumor.^24^ Therefore, elective ND is the only reliable way for identifying extracapsular spread in these patients and intensifying treatment, if necessary. However, various imaging size criteria are available to detect abnormal nodes, but none are specific enough to differentiate ENE.^36^

Performing an initial ND approach might be a strategy to control regional disease by removing necrotic or hypoxic bulky neck nodes before CTX‐RT.^37^ Nevertheless, regional and distant progression can still occur in these patients, suggesting that metastatic potential is intrinsic to the initial nodal stage. Furthermore, in patients with resectable N3 (AJCC 7th) disease, ND has improved locoregional control but implies surgical morbidity. It has also proven to provide no survival benefit, as the risk of metastasis is comparable to that of patients with unresectable disease.^38^

Considering TDs a potential adverse high‐risk factor, adjuvant intensification therapy would be recommended. However, even with this approach, the prognosis remains poor. Prabhu et al. observed significantly worse OS in 15 patients with soft tissue metastasis (corresponding to grade 4 of the Lewis scale) despite the association of CTX with RT.^22^, ^27^ Their study reported a two‐year OS of 38.1%, similar to the three‐year OS seen in patients with TDs in this study (38.4%). Moreover, a recent meta‐analysis regarding systemic treatment in nasopharynx carcinoma concluded that combining induction or adjuvant CTX with concomitant CTX‐RT improved survival rates.^39^ Given the high incidence of DM in the presence of TDs, patients may benefit from sequential CTX.^40^

While this study provides valuable insights, there are certain limitations that should be acknowledged. Firstly, the sample size is relatively small, and the retrospective design lacks randomized patient groups. In addition, our results may have been affected by potential confounding factors that were not included in the analysis, such as surgical margins, vascular invasion, nerve invasion, and histopathologic grade. These clinical and pathological factors could have an impact on the prognostic significance of ENE and TDs in HNSCC, and should be considered in future research. Nevertheless, the strength of this study lies in the analysis of an equally staged, treated, and monitored population.

To our knowledge, this study is the first to examine the AJCC's proposal to distinguish pN2a‐ENE from pN3b‐ENE, as well as the prognostic implications of TDs, highlighting the need to consider TDs as a distinct entity. However, larger studies are necessary to identify the most effective treatment for these clinical scenarios and to validate our results. At present, the optimal therapeutic approach remains unclear.

## 5 CONCLUSIONS

TDs in HNSCC were present in 29.6% of patients, and their presence was associated with a lower 3‐year OS rate compared to ENE cases, despite intensified adjuvant therapy with surgery followed by CTX‐RT. The results confirm a better prognosis for pN2a‐ENE compared to pN3b‐ENE, and for pN3b‐ENE compared to TDs. Furthermore, the findings of this study suggest that TDs could be an important prognostic indicator in HNSCC, and their inclusion in the TNM staging system could provide a more accurate stratification of different risk groups. Further studies with larger sample sizes are needed to confirm these results and to evaluate the impact of TDs on treatment strategies and patient outcomes in HNSCC.

## AUTHOR CONTRIBUTIONS

**Leyre González‐Vallejo**: Design, data acquisition, analysis and interpretation, critical revision, final approval. **Javier Blanco‐Sainzdelamaza**: Design, data acquisition, statistical analysis and interpretation, drafting, critical revision, final approval. **Arrate Querejeta‐Ayerra**: Critical revision, final approval. **Carlos Chiesa‐Estomba**: critical revision, final draft approval.

## CONFLICT OF INTEREST STATEMENT

The authors have stated explicitly that there are no conflicts of interest in connection with this article.

## Data Availability

Data available on request from the authors.
